# Pain and Oral-Health-Related Quality of Life in Orthodontic Patients During Initial Therapy with Conventional, Low-Friction, and Lingual Brackets and Aligners (Invisalign): A Prospective Clinical Study

**DOI:** 10.3390/jcm9072088

**Published:** 2020-07-03

**Authors:** Laura Antonio-Zancajo, Javier Montero, Alberto Albaladejo, Maria Dolores Oteo-Calatayud, Alfonso Alvarado-Lorenzo

**Affiliations:** 1 Faculty of Medicine, University of Salamanca, Avenida Alfonso X el Sabio s/n, 37007 Salamanca, Spain; javimont@usal.es (J.M.); albertoalbaladejo@hotmail.com (A.A.); alfonsoalvaradolorenzo@gmail.com (A.A.-L.); 2 School of Dentistry University Complutense of Madrid, Plaza Ramón y Cajal, s/n, 28040 Madrid, Spain; mdoteo@ucm.es

**Keywords:** orthodontics, pain, oral quality of life, low-friction brackets, lingual orthodontics, Invisalign, oral health

## Abstract

The aim of this study was to compare pain and its relationship with the oral quality of life of patients with different types of orthodontic appliances: conventional and conventional low-friction brackets, lingual brackets, and aligners. A prospective clinical study was carried out with a sample size of 120 patients (54 men, 66 women) divided into 4 groups of 30 patients each. The modified McGill questionnaire was used to measure pain at 4, 8, and 24 h and 2, 3, 4, 5, 6, and 7 days after the start of treatment, and the Oral Health Impact Profile-14 (OHIP-14) questionnaire was used to measure the oral-health-related quality of life (OHRQoL) in the first month of treatment. The maximum peak of pain was obtained between 24 and 48 h of treatment. It was found that patients in the lingual orthodontic group described lower levels of pain at all times analyzed, and their scores in the total OHIP-14 indicated less impact on their oral quality of life (1.3 ± 1.2, *p* < 0.01) compared with the other groups analyzed. There was little difference with the aligners group (Invisalign) (1.7 ± 1.9, *p* < 0.01). The technique used influences the pain and quality of life of patients at the start of orthodontic treatment.

## 1. Introduction

Regarding orthodontic treatment, around 90% of patients state that pain and discomfort are the main drawbacks [1]. When faced with a high pain level, patients may interrupt and/or end treatment [2]. It is considered that 39% of patients experience pain and/or discomfort after each check-up appointment for their appliance, and the type of pain is usually mild/moderate (56–69%), of short duration (45% lasted less than two days), during mastication (82%), and not spontaneous (associated with masticatory function, when clenching teeth, or while brushing) [3,4,5].

During orthodontic treatment, different forces are applied through the brackets and arches that cause tooth movement in the alveolar bone [6]. Patients report two types of pain: immediate pain related to periodontal compression and delayed pain related to the inflammatory response [1]. This increase, or pain peak, at the start of treatment occurs mainly 24 h after the placement of elastics and/or fixed multibracket appliances [7,8]. Then, it decreases until reaching minimum values after 7 days [8,9,10]. According to a study carried out by Rakhsan, general discomfort occurs 65.7% of the time, and 34.3% of the time, localized discomfort occurs. The pain is of moderate intensity during active chewing of hard and fibrous foods and of mild intensity during chewing of soft foods and brushing [4,5].

Pain is a subjective response, showing great individual variation. It depends on factors such as individual threshold, magnitude of applied force, emotional/stress status, cultural differences, and previous pain experiences [11]. Regarding gender, most studies have concluded that there are no statistically significant differences among orthodontic appliance users [8,12,13].

Therefore, the pain that patients experience and the discomfort associated with orthodontic treatment will negatively influence the quality of a patient’s oral life [8,14,15]. One of the justifications for orthodontic treatment is based on ultimately improving the health-related quality of life. Therefore, studying the OHRQoL (Oral Health in Relation to Quality of Life) in our orthodontic patients provided us with valuable information on their needs and also treatment results [16].

According to most authors, self-ligating brackets produce less pain and have less impact on the oral quality of life than conventional fixed orthodontic treatment [8,12,13,17,18]. Regarding lingual orthodontics, most authors consider that there is greater pain and impact on function (speech and chewing) in these patients compared with users of conventional orthodontics; however, aesthetics are improved [19,20,21]. This improvement in the perception of aesthetics and comfort by patients has also been observed in users of the Invisalign system, with a lower perception of pain from the second day onwards when compared with patients treated with conventional orthodontics [14,22].

In this study, we evaluated how four different types of orthodontic appliances affected dental pain and its intensity during the first week and the impact on quality of life during the first month of treatment. The null hypotheses were as follows: There is no significant difference in pain and OHRQoL in orthodontic patients with conventional, low-friction, and lingual brackets and aligners. The alternate hypotheses were as follows: There is a significant difference in pain and OHRQoL in orthodontic patients with conventional, low-friction, and lingual brackets and aligners.

## 2. Experimental Section

### 2.1. Ethics Approval and Patient Consent

The research project was approved by the Bioethics Committee of the University of Salamanca (USAL_16/060). A prospective one-month follow-up clinical study was performed after the placement of four different orthodontic appliances: conventional, low-friction, and lingual brackets and orthodontic aligners (Invisalign). The guidelines established by the Declaration of Helsinki for research on humans were followed. Prior to participation in the study, patients were told that participation was voluntary, and the treatment protocol was explained to them. Furthermore, they were asked for their written consent.

### 2.2. Sample Size Calculation and Participants

Sample size was calculated using Raosoft’s online sample size calculator (Raosoft Inc., Seattle, WA, USA). Using a 5% margin of error and a 95% confidence level, the target sample size was determined to be 120 patients, including a 10% dropout.

This one-month prospective clinical study was performed on a total sample of 120 patients (55% female and 45% male), divided into four groups of 30 individuals each. This sample size was similar to that used in other previously published studies [12,23,24]. The first group (Conventional—CON) consisted of orthodontic patients with conventional brackets (Victory Series^®^, 3M, Rogers, Arkansas, USA). The second group (Low friction—LF) was treated with conventional low-friction brackets (Synergy^®^, Rocky Mountain Orthodontics, Denver, Colorado, USA). The third group (Lingual orthodontics—LO) wore multibracket fixed lingual brackets (STB^®^ from Ormco, Orange, California, USA). The slot dimension of the brackets described above was 0.018" × 0.025" in bracket groups. Finally, the fourth group (Invisalign™—INV) used an aligner system (Invisalign^®^ from Align Technology Inc., San José, California, USA), following Invisalign’s own treatment initiation protocol. The arches used were a Cooper NiTI arch of 0.014" in the CON and LF groups and a universal preform Cooper NiTI arch of 0.013" with individual 0.10 in. metal wires in the LO group, in accordance with the STB^®^ technique protocol described by Dr. Scuzzo [25]. All of them were linked by 0.10 in. metal wires.

The inclusion criteria were as follows:

- Patients between 18 and 40 years of age and with permanent dentition;
- Without previous orthodontic treatment;
- No previous extractions except third molars;
- Dental bone discrepancy between –2 and –6 mm in both arches;
- Good oral health without caries or periodontal disease;
- Skeletal class I or mild classes II and III (ANB 0°–5°) [26].

The exclusion criteria were as follows:

- Patients with deciduous teeth or in the process of dental replacement;
- Patients in need of orthodontic surgical treatment or dental extractions due to treatment;
- Patients with systemic diseases;
- Patients with medication that influences pain perception (analgesics, antidepressants, and/or anticonvulsants);
- Severe malformations;
- Anatomy of the lingual side that would prevent lingual brackets being cemented in the lingual orthodontic group.

Before starting the study, we performed a periodontal evaluation. All patients began the study in good oral health.

### 2.3. Study Design

After the placement of the appliances, the patients were given a visual analog scale (VAS) based on the McGill modified questionnaire to reflect the pain suffered and another to analyze their quality of life [27,28]. Before they filled them in, we made sure that they understood the instructions. 

Pain intensity was measured using VAS with values ranging from 0 (no pain) to 10 (maximum pain) on a 10 cm long line with different points in time after starting orthodontic treatment: 4 h (T4h), 8 h (T8h), 24 h (T1), 2 days (T2), 3 days (T3), 4 days (T4), 5 days (T5), 6 days (T6), 7 days (T7), and from the 7th day (T18) [29,30].

Furthermore, after the first month of treatment, the patients had to complete the Oral Health Impact Profile-14 (OHIP-14) questionnaire to analyze the level of impact on their oral quality of life [31,32]. The frequency of occurrence of problems/dysfunctions in 14 items following orthodontic treatment was determined on a 5-point Likert scale (0 = never, 1 = almost never, 2 = occasionally, 3 = quite often, 4 = very often). This questionnaire has been validated for the Spanish population by Montero et al., and it has been used to measure the impact of different interventions [32].

The total impact on quality of life was calculated as the sum of the items, the frequency level of which was occasional or more frequent in order to obtain a pure, quantitatively derived variable (OHIP-Total) that reflected each patient’s global well-being. This same strategy was used to compute the impact on each of the 7 domains of the OHIP (functional limitation, pain, mental discomfort, physical disability, mental disability, social disability, and handicap), each consisting of two items.

### 2.4. Statistical Analysis

To describe the studied population, the mean and standard deviation were used as quantitative variables, and the sampling distribution (number of patients and the corresponding percentage) was determined for the nominal and ordinal data. To compare the groups with respect to quantitative variables, the ANOVA *F*-test was used. If it was statistically significant, a comparison between groups was made using the Bonferroni post hoc test. The Chi Square test was performed to compare two or more nominal or ordinal distributions. A *p*-value of less than 0.05 (*p* < 0.05) was established to declare a difference as statistically significant, and a *p*-value of less than 0.01 (*p* < 0.01) was used to consider the result highly significant. In addition, we considered the results with *p*-values between 0.06 and 0.10 as tending toward statistical significance. SPSS v. 20 software (SPSS Inc., Chicago, IL, USA) was used to analyze the data.

## 3. Results

### 3.1. Characteristics of the Participants

The mean age of the sample was 30.0 ± 7.5 years. Of the 120 patients, 66 were women (55%) and 54 were men (45%). The degree of crowding was measured as the skeletal discrepancy prior to treatment; no statistically significant differences were observed (Table 1).

### 3.2. Pain Analysis

After the ANOVA test analysis of the results, statistically significant differences (*p* < 0.01) in the pain level were found at 4 h after treatment according to the VAS scale. The group with the most pain at this time point was the conventional bracket group (3.8 ± 2.3), with similar results to the Invisalign group (2.8 ± 2.5), and the lingual bracket group had the least pain (1.7 ± 2.5) next to the low-friction group (2.0 ± 1.9). After 48 h and also with a *p* < 0.01, a higher level of pain was observed in low-friction patients (5.8 ± 1.9), without differences with the conventional group, while those with lingual brackets continued to show the lowest level of pain (2.7 ± 2.1). This trend continued until T7 (Table 2).

At 24 h and 7 days after treatment, we also found statistically significant differences (*p* < 0.05), with the low-friction bracket group presenting the highest level of pain (5.6 ± 2.0 and 0.8 ± 1.6, respectively) and the lingual bracket group presenting the least pain (4 ± 1.9 and 0.04 ± 0.2). However, after 8 h of treatment, a value of *p* = 0.06 was achieved, a value that is almost statistically significant, with greater pain occurring in the conventional group (4.7 ± 2.3) and less pain occurring in the lingual bracket group (3.3 ± 1.9) (Table 2).

**Figure 1:**
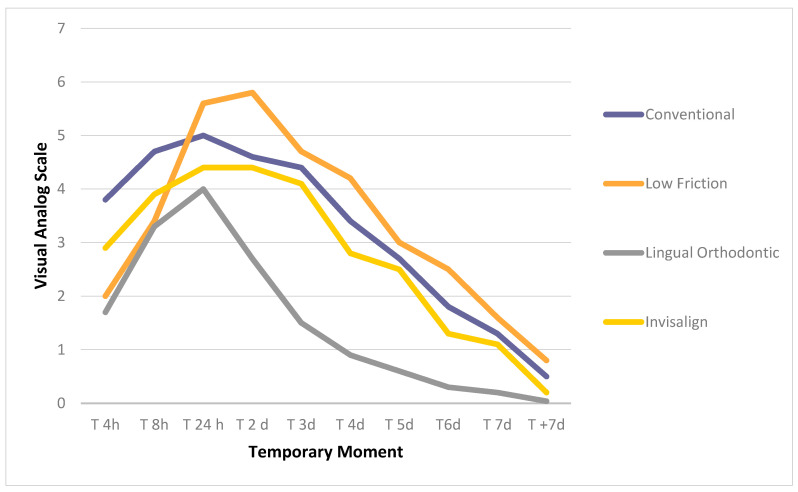
Pain analysis according to the visual analog scale (VAS).

The maximum pain peak was reached 24 h after the start of treatment in the conventional (5.0 ± 2.7), lingual (4 ± 1.9), and Invisalign (4.4 ± 2.1) groups and at 48 h in the low-friction group (5.8 ± 1.9). After this time, the pain began to decrease gradually (Table 2, Figure 1).

### 3.3. Analysis of the Quality of Life Related to Oral Health

When we analyzed the impact on the quality of life domains in relation to oral health (Table 3), we observed a greater negative impact on pain levels (1.6 ± 0.6, *p* < 0.01) and physical disability (0.6 ± 0.7, *p* < 0.01) in the conventional group, with the lingual group having the smallest impact on pain (0.8 ± 0.8) and the Invisalign group having the lowest effect on physical disability (0.03 ± 0.2). There were no differences with the low-friction group in both categories.

On the other hand, we found higher scores in the low-friction group, with a greater negative impact compared with the lingual bracket group at the levels of psychological discomfort (LF: 1.3 ± 0.8, LO: 0.03 ± 0.2, *p* < 0.01), psychological disability (LF: 0.8 ± 0.9, LO: 0.0 ± 0.0, *p* < 0.01), and social disability (LF: 0.3 ± 0.5, LO: 0.0 ± 0.0, *p* < 0.05), as well as for the total impact scores (LF: 4.5 ± 2.8, LO: 1.3 ± 1.2, *p* < 0.01). The values found in the Invisalign group in these dimensions were similar with respect to the lingual group (Table 3).

We did not find statistically significant differences in terms of functional limitations among the four groups analyzed, although, as we can see in Table 3, patients with low-friction and Invisalign appliances reported a greater negative impact than patients in the lingual bracket groups (Table 3).

## 4. Discussion

This study attempted to analyze and compare pain and quality of life in patients with different types of appliances during the initial period of use. In the literature, there are numerous studies that have analyzed pain in orthodontics [12,15,23] and, above all, that have compared conventional brackets with low-friction brackets [23,24]. Fewer studies have analyzed the quality of life of patients undergoing orthodontic treatment [17,33]. Of these studies, none have analyzed the effects of four different orthodontic techniques, taking into account different quantitative and ordinal variables using a standardized questionnaire. Some of the limitations of our study include the brevity of the follow-up time, and the fact that influential variables, such as the level of stress or anxiety prior to treatment, an individual’s pain threshold, or previous experiences of pain in the dental office, were not taken into account for the analysis of pain perception. Regarding oral quality of life, cultural differences and the social environment that surrounds patients were not taken into account [2,11,34].

Most of the studies found used brackets with a 0.022" slot groove [35,36]. Other studies did not specify the size of the slot used [24,37,38]. None of them took the slot size into account when assessing the level of pain or the quality of life. In orthodontics, 0.018" or 0.022" slots can be used interchangeably. Here, we decided to use a 0.018" slot in the three groups of brackets so that this variable was homogeneous to avoid bias.

In the literature, we found that the maximum pain peak occurred 24 h after the placement of the orthodontic appliances, and pain decreased throughout the first week of treatment [8,9,23,39]. We observed a similar pain pattern in the conventional, lingual, and Invisalign groups, but the pain peak occurred at 48 h in the conventional low-friction group (Figure 1). For the assessment of the degree of pain, most previous studies, like ours, used the VAS scale, since it allows the pain of the same patient to be quantified at different time points to observe its evolution [29,30]. The reliability of the VAS for pain measurements was confirmed by Revill et al. [40] and was shown to be adequate for repeated measurements within the same individual [41].

The different comparative studies between orthodontic techniques have mostly used conventional orthodontics as a control group. They have observed how low-friction patients present with less pain during the first phases of treatment [23,34]. In our study, we found the same results: the conventional group presented an average VAS score of 3.8 ± 2.3 at 4 h versus the low-friction group’s score of 2.0 ± 1.9. After 24 h, the order changed, with the conventional low-friction group presenting the highest pain level (Table 2).

Patients in the lingual orthodontic group presented a lower level of pain than patients who underwent the other techniques analyzed at all time points. The greatest discrepancies between groups were observed at 48 h with the conventional group (CON: 4.6 ± 2.5, LO: 2.7 ± 2.1), at 3 days with the Invisalign group (INV: 4.1 ± 2.5, LO: 2.3 ± 1.5), and at 4 days with the low-friction group (LF: 4.2 ± 2.4, LO: 0.9 ± 1.3) (Table 2). In the literature, we found comparative studies of conventional and lingual brackets that showed different results. While some authors stated that there are no statistically significant differences between conventional and lingual patients [42,43], most of the consulted authors found that lingual orthodontic patients experienced more pain [20,44,45,46].

In comparative studies between conventional and Invisalign appliances, it was observed that the pain in patients who underwent the two techniques was similar during the first two days of treatment and greater in conventional treatments from the third to the fourth days [9,47]. Our results show that patients suffered a greater degree of pain with conventional brackets, although there was not much difference between the two groups at all points studied. The greatest difference was found after 4 h of analysis (CON: 3.8 ± 2.3, INV: 2.9 ± 2.5) (Table 2).

Other authors observed that Invisalign (first 24 h) and lingual (after 2 days of treatment) patients were associated with more dental pain compared with conventional orthodontic treatment [45]. These results are in contrast to those obtained in our study, where we observed that the lingual group presented less pain from the start of treatment and throughout all time points according to the VAS scale. This difference in results may be due to the fact that, in that study [45], a 0.022" slot was used for conventional orthodontics and a 0.018" slot was used in lingual orthodontics; in our study, 0.018" was used in all types of brackets.

According to the literature published in recent years, there does not appear to be a statistically significant difference in pain with the different arches analyzed [35,48,49] or between the use of different arch sequences and greater presence of pain [37,50]. It is considered that there were no differences in the degree of pain experienced by the patients due to the fact that different arch thicknesses were used in our study (0.013" CuNiTi in lingual brackets and 0.014" Nitinol in conventional and low friction). However, we think that it would be necessary to carry out further studies comparing arches with 0.013" and 0.014" thicknesses to see whether or not there are differences in pain, since the majority of published studies have compared arches of 0.014" or thicker. 

Several questionnaires have been used to assess the oral quality of life in orthodontic patients. In this study, the OHIP-14 questionnaire was chosen because it is the most widely used in the consulted literature and is easy to complete by patients [18,33,51]. In 2009, Montero et al. verified the usefulness of this questionnaire in the Spanish adult population [32].

Low-friction brackets have less impact on quality of life than conventional brackets [17,18,52]. We found that patients in the low-friction group had the greatest total impact on the oral quality of life (4.5 ± 2.8), while the lingual orthodontic group (1.3 ± 1.2) had the least impact, with statistically significant differences between the groups (ANOVA *F*: 16; gl: 3; *p*-value: 0.00) (Table 3).

Classically, it has been considered that, due to the morphology and placement of the brackets, lingual appliances lead to greater pain and discomfort for the patients, negatively influencing their oral quality of life (speech, chewing, pronunciation, etc.) [43,44,53]. Likewise, it has been considered that appliances with aligners improve patients’ acceptance of the treatment, with a reduction in pain and discomfort as well as an aesthetic improvement [9,14,54]. Patients wearing aligners were found to have less pain, less discomfort during chewing, and less impact on social and psychological disability during the first week of analysis compared with those with conventional or low-friction braces. No statistically significant differences in pronunciation were found [55].

In our analysis, we observed lower impact values for all quality-of-life domains in the lingual bracket group (Table 3). We did not find statistically significant differences in terms of functional limitation (speech and chewing); however, other authors consulted found that speech and pronunciation were affected by lingual appliances [19,20,21,46,56]. A total 23.3% of patients with lingual brackets were found to still have difficulties with their pronunciation after three months [46]. These authors considered that pronunciation is affected because language requires a specific position to pronounce each phoneme and this position is modified by the placement of the lingual brackets. We consider, like Slater in his 2013 article [56], that the new design of lingual brackets (smaller and more comfortable) reduces the negative impact on this quality-of-life domain.

The differences found in other studies [45,55] may be due to the heterogeneity in their samples (large skeletal discrepancies, different types of malocclusions, etc.). With this study, we tried to homogenize the patients in the four treatment groups in terms of number, type, and degree of malocclusion and by not including treatment with extractions or surgical cases. We believe that the inclusion of patients with large differences in their skeletal discrepancy or the non-specification of it in the inclusion criteria made it impossible for us to compare the results of the three studies, since the assessment of the oral quality of life, and especially pain, can be greatly influenced by these parameters.

One of the limitations when conducting this study was the achievement of a homogeneous sample with respect to sex or age, since most patients who choose more aesthetic techniques (lingual and Invisalign) or who choose to receive orthodontic treatment in general are women and adults. In the literature, we found that sex does not influence pain perceived by adult patients and that age influences the perceived pain to a greater extent in adolescents and not in adults [8,12,13]. In the future, we want to carry out a randomized study with greater homogeneity between the samples of each group, although it is difficult to get patients to use fixed appliances when looking for aesthetic treatments.

To the best of our knowledge, this is the first study to compare four different orthodontic techniques. Our main objective was to analyze pain and the influences of different orthodontic techniques on improving the oral health and quality of life of our patients during treatment. In the future, we intend to expand the number of participants in the sample, equalize the percentage of women and men, and include other types of orthodontic appliances for comparison.

## 5. Conclusions

The maximum peak of pain was reached between the first 24 h (conventional, lingual, and Invisalign) and 48 h (low friction) after treatment, and then, it decreased to near zero at the end of the first week of orthodontic treatment. Lingual bracket patients presented the lowest level of pain at all time points and had the least negative impact, with statistically significant differences in terms of pain, psychological discomfort, psychological disability, social disability, and total OHIP-14 scores. The results obtained in this study showed a slightly higher quality of life in patients using aligners than in lingual patients, with no large discrepancies between groups. However, there were great differences between the conventional and low-friction groups.

Based on the results of this study, we reject the null hypothesis and accept the alternate hypothesis that there is significant difference in pain and OHRQoL in orthodontic patients with conventional, low-friction, and lingual brackets and aligners.

## Figures and Tables

**Table 1:** Demographic characteristics of participants (*n* = 120).

			Conventional Brackets (*n* = 30)	Low-Friction Brackets (*n* = 30)	Lingual Brackets (*n* = 30)	Invisalign (*n* = 30)
**Age (Years)**	**Mean**		24.7	28	33.8	33.4
	**SD**		4.1	9.7	8.2	5.1
**Sex**	**Men**	**N**	13	12	13	16
		**%**	43.3	40	43.3	53.3
	**Women**	**N**	17	18	17	14
		**%**	56.7	60	56.7	46.6
**Dental Bone Discrepancy**	**Upper**	**Mean**	−3.1	−3.1	−3.0	−2.6
		**SD.**	1.0	0.7	1.6	1.6
	**Lower**	**Mean**	−3.3	−2.7	−3.4	−2.6
		**SD.**	1.3	1.2	1.5	1.7

SD: standard deviation.

**Table 2:** Comparison of dental pain on the visual analog scale between groups at the different evaluation times (*n* = 120).

Time	Conventional Brackets (*n* = 30)		Low-Friction Brackets (*n* = 30)		Lingual Brackets (*n* = 30)		Invisalign (*n* = 30)	
	Mean	SD	Mean	SD	Mean	SD	Mean	SD
4 h (T4h) **	3.8 ^A^	2.3	2.0 ^B,C^	1.9	1.7 ^C^	2.3	2.9 ^A,B,C^	2.5
8 h (T8h)	4.7	2.3	3.4	2.2	3.3	1.9	3.9	2.3
24 h (T1) *	5.0 ^a,c^	2.7	5.6 ^a^	2.0	4 ^c^	1.9	4.4 ^a,c^	2.1
2 days (T2) **	4.6 ^A^	2.5	5.8 ^A^	1.9	2.7 ^C^	2.1	4.4 ^A^	2.4
3 days (T3) **	4.4 ^A^	3.0	4.7 ^A^	2.3	1.5 ^C^	1.7	4.1 ^A^	2.5
4 days (T4) **	3.4 ^A^	2.7	4.2 ^A^	2.4	0.9 ^C^	1.3	2.8 ^B^	2.1
5 days (T5) **	2.7 ^A^	2.4	3.0 ^A^	2.5	0.6 ^C^	1.2	2.5 ^A^	1.9
6 days (T6) **	1.8 ^A^	1.9	2.5 ^A^	2.2	0.3 ^C^	0.9	1.3 ^B^	1.7
7 days (T7) **	1.3 ^A,B^	1.6	1.6 ^B^	2.1	0.2 ^A^	0.8	1.1 ^A,B^	1.6

* Statistically significant results (*p* < 0.05). ** Statistically significant results (*p* < 0.01). Different superscript letters in the rows indicate in which groups the significant differences occurred with Bonferroni’s post hoc tests. A = *p* < 0.01 vs. CON; a = *p* < 0.05 vs. CON. B = *p* < 0.01 vs. LF; C = *p* < 0.01 vs. LO; c = *p* < 0.05 vs. LO.

**Table 3:** Comparison of the impact on the quality-of-life domains among the treatment groups (*n* = 120).

Domains	Conventional Brackets (*n* = 30)		Low-Friction Brackets (*n* = 30)		Lingual Brackets (*n* = 30)		Invisalign (*n* = 30)	
	Mean	SD	Mean	SD	Mean	SD	Mean	SD
Functional limitation	0.4	0.6	0.6	0.8	0.3	0.5	0.5	0.7
	ANOVA *F*: 1.5; fd: 3; *p*-value: 0.22							
Physical pain **	1.6 ^A^	0.6	1.3 ^A,C^	0.8	0.8 ^C^	0.8	0.9 ^A,C^	0.7
	ANOVA *F*: 7.7; fd: 3; *p*-value: 0.00							
Psychological discomfort **	0.8 ^A^	0.9	1.3 ^B^	0.8	0.0 ^C^	0.2	0.2 ^C,D^	0.4
	ANOVA *F*: 24.4; fd: 3; *p*-value: 0.00							
Physical disability **	0.6 ^A^	0.7	2 ^B^	0.5	0.1 ^B^	0.4	0.0 ^B^	0.2
	ANOVA *F*: 9.7; fd: 3; *p*-value: 0.00							
Psychological disability **	0.3 ^A,C^	0.5	0.8 ^B^	0.9	0.0 ^C^	0.0	0.1 ^A,C,D^	0.4
	ANOVA *F*: 12.5; fd: 3; *p*-value: 0.00							
Social disability *	0.1 ^a,c^	0.4	3 ^a^	0.5	0.0 ^c^	0.0	0.1 ^a,c^	0.7
	ANOVA *F*: 3.1; fd: 3; *p*-value: 0.03							
Handicap	0.0	0.0	0.1	0.2	0.0	0.0	0.1	0.4
	ANOVA *F*: 0.7; fd: 3; *p*-value: 0.53							
Total OHIP **	3.8 ^A^	2.1	4.5 ^A,B^	2.8	1.3 ^C^	1.2	1.7 ^C,D^	1.9
	ANOVA *F*: 16; fd: 3; *p*-value: 0.00							

* Statistically significant result (*p* < 0.05). ** Statistically significant result (*p* < 0.01). Different superscript letters in the rows indicate in which groups the significant differences occurred with Bonferroni’s post hoc tests. A = *p* < 0.01 vs. CON; a = *p* < 0.05 vs. CON. B = *p* < 0.01 vs. LF; C = *p* < 0.01 vs. LO; c = *p* < 0.05 vs. LO. D = *p* < 0.01 vs. INV.
